# Letter to the Editor in response to “COVID-19: desperate times call for desperate measures”

**DOI:** 10.1186/s13054-020-03152-6

**Published:** 2020-07-10

**Authors:** J. Geoffrey Chase, Yeong-Shiong Chiew, Bernard Lambermont, Philippe Morimont, Geoffrey M. Shaw, Thomas Desaive

**Affiliations:** 1 Department of Mechanical Engineering, University of Canterbury, Christchurch, New Zealand; 2 School of Engineering, Monash University, Bandar Sunway, Malaysia; 3 Department of Intensive Care, University of Liège hospital, Liège, Belgium; 4 Department of Intensive Care, Christchurch Hospital, Christchurch, New Zealand; 5 GIGA-In Silico Medicine, University of Liège, Liège, Belgium

We have read with attention the commentary from von Düring et al. questioning our research letter on safe doubling of ventilator capacity recently published in *Critical Care* [1]. Our publication starts with a quote of our own: “The best way to ventilate two patients on a single ventilator is simply not to do it,” and we believe that unfortunately, von Düring et al. took this as the conclusion of the report.

In fact, we argue for the idea of ventilating two patients with a single ventilator, not against. This is a serious misunderstanding of our work proposing a safe way to ventilate two patients at once, so as von Düring et al. note, a second life can be saved.

We proposed a safe, innovative method based on a simple *in-series* (one-after-the-other) breathing circuit (Fig. 1). It directly addresses the limitations of shared, in-parallel (both-together) breathing listed in the SCCM statement [2]. It permits individual PEEP settings and driving pressures, and volume-controlled or pressure-controlled ventilation of patients with different lung compliance, because each patient breathes separately.

**Fig. 1:**
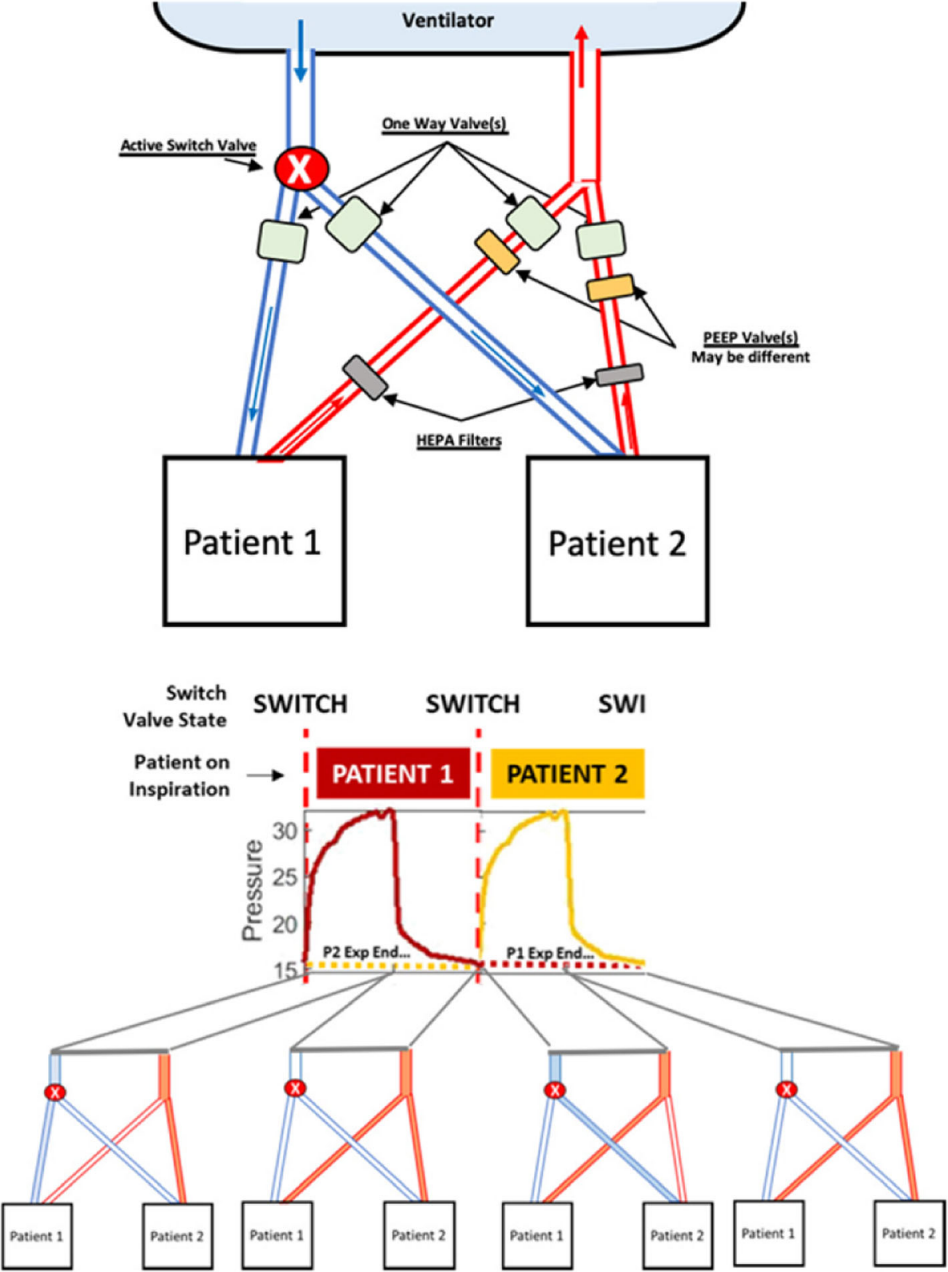
In-series breathing setup and concept [1]

While we agree with von Düring et al. when they say “The objective of ventilator sharing is to save a second life by buying time to find a second ventilator” [3], but we believe it is practically incompatible with their suggestion of reserving it for patients with the same gender, similar IBW and lung mechanics, who are sedated and paralyzed. When ICU clinicians are faced with a decision to ventilate two patients at once, or deny care to one, they will not start analyzing all of their patients’ lung mechanics to find the matching pairs. Our in-series approach obviates this need.

So yes, we believe our in-series setup is a *safe last resort proposal* for last resorts, rather than a *desperate measure*.

## Data Availability

Not applicable
